# Tau plasma levels in subjective cognitive decline: Results from the DELCODE study

**DOI:** 10.1038/s41598-017-08779-0

**Published:** 2017-08-25

**Authors:** Stephan Müller, Oliver Preische, Jens C. Göpfert, Viviana A. Carcamo Yañez, Thomas O. Joos, Henning Boecker, Emrah Düzel, Peter Falkai, Josef Priller, Katharina Buerger, Cihan Catak, Daniel Janowitz, Michael T. Heneka, Frederic Brosseron, Peter Nestor, Oliver Peters, Felix Menne, Carola G. Schipke, Anja Schneider, Annika Spottke, Klaus Fließbach, Ingo Kilimann, Stefan Teipel, Michael Wagner, Jens Wiltfang, Frank Jessen, Christoph Laske

**Affiliations:** 1 Section for Dementia Research, Hertie Institute for Clinical Brain Research and Department of Psychiatry and Psychotherapy, University of Tübingen, Tübingen, Germany; 2 German Center for Neurodegenerative Diseases (DZNE), Tübingen, Germany; 3 Natural and Medical Sciences Institute at the University of Tübingen, Reutlingen, Germany; 4 German Center for Neurodegenerative Diseases (DZNE), Bonn, Germany; 5 German Center for Neurodegenerative Diseases (DZNE), Magdeburg, Germany; 6 German Center for Neurodegenerative Diseases (DZNE), Munich, Germany; 7 German Center for Neurodegenerative Diseases (DZNE), Berlin, Germany; 8 Department of Neuropsychiatry, Charité - Universitätsmedizin Berlin & Berlin Institute of Health, Berlin, Germany; 9 Institute for Stroke and Dementia Research (ISD), Klinikum der Universität München, Munich, Germany; 10 Department of Neurology, University Hospital Bonn, Bonn, Germany; 11 Department for Neurodegenerative Diseases and Gerontopsychiatry, University Hospital Bonn, Bonn, Germany; 12 German Center for Neurodegenerative Diseases (DZNE), Rostock, Germany; 13 Department of Psychiatry, University Hospital Bonn, Bonn, Germany; 14 German Center for Neurodegenerative Diseases (DZNE), Göttingen, Germany; 15 Department of Psychiatry, Medical Faculty, University of Cologne, Cologne, Germany; 16 Charité – Universitätsmedizin Berlin, Institute of Psychiatry and Psychotherapy, CBF, Berlin, Germany; 17 Charité – Universitätsmedizin Berlin, Institute of Neuropathology, Berlin, Germany; 18 Department of Psychiatry and Psychotherapy, University Medical Center Goettingen (UMG), Goettingen, Germany

## Abstract

Previous studies have demonstrated increased tau plasma levels in patients with Alzheimer’s disease (AD) and mild cognitive impairment (MCI) due to AD. Much less is known whether increased tau plasma levels can already be detected in the pre-MCI stage of subjective cognitive decline (SCD). In the present study we measured tau plasma levels in 111 SCD patients and 134 age- and gender-matched cognitively healthy controls participating in the DZNE (German Center for Neurodegenerative Diseases) longitudinal study on cognition and dementia (DELCODE). Tau plasma levels were measured using ultra-sensitive, single-molecule array (Simoa) technology. We found no significant different tau plasma levels in SCD (3.4 pg/ml) compared with healthy controls (3.6 pg/ml) after controlling for age, gender, and education (p = 0.137). In addition, tau plasma levels did not correlate with Aβ42 (r = 0.073; p = 0.634), tau (r = −0.179; p = 0.240), and p-tau181 (r = −0.208; p = 0.171) cerebrospinal fluid (CSF) levels in a subgroup of 45 SCD patients with available CSF. In conclusion, plasma tau is not increased in SCD patients. In addition, the lack of correlation between tau in plasma and CSF in the examined cohort suggests that tau levels are affected by different factors in both biofluids.

## Introduction

Brain deposition of neurofibrillary tangles (NFTs) composed of hyperphosphorylated tau is a hallmark of Alzheimer’s disease (AD) pathology^1^. Several tau tracers for positron emission tomography (PET) imaging including 18F-TH523 have been developed over the past few years^2^. As in the brain itself, tau levels in the cerebrospinal fluid (CSF) were found to be increased in AD patients^3^. Therefore, CSF tau provides a useful marker of tau pathology. Several tau-based therapeutic approaches are currently investigated. Thus, detection of tau levels holds enormous potential for both early diagnosis of AD and monitoring of disease-modifying therapeutics.

An ideal test for diagnosis and monitoring of disease-modifying therapeutics in AD should be applicable with the lowest possible risk, easy and fast to perform and cheap. A blood test would fulfil all of these conditions in contrast to PET neuroimaging (expensive; exposure to ionizing radiation) and CSF analysis (invasive). Indeed, several previous studies have demonstrated increased tau plasma levels in patients with AD^4–6^, in a group of patients with AD and mild cognitive impairment (MCI) due to AD^7^ and in MCI due to AD^4^. Much less is known whether increased tau plasma levels can already be detected in the pre-MCI stage of subjective cognitive decline (SCD). Only one recent study examined this association and failed to demonstrate increased tau plasma levels in SCD patients^5^.

SCD is actually considered to be associated with an increased likelihood of future cognitive impairment and dementia, especially in those cases with worry about memory^8–10^. According to the suggestions made by the Working Group of the Subjective Cognitive Decline Initiative (SCD-I)^11^, SCD is defined as personal complaints about ones’ cognitive state in the absence of objective cognitive impairment. This definition of SCD was also used in the present study.

The aim of the present study was to examine tau plasma levels in SCD patients and healthy controls (HC) participating in the DELCODE study and to investigate the association with clinical parameters (age, gender, and education), neuropsychological parameters (Mini-Mental-State Examination [MMSE], Clinical Dementia Rating [CDR], and logical memory subtest of the Wechsler Memory Scale) and biochemical markers of AD (CSF levels of beta-amyloid 42 [Aβ42], tau and phosphorylated tau181 [p-tau181]).

## Materials and Methods

### Subjects

111 SCD patients and 134 age- and gender-matched cognitively healthy controls were included in the study (Table 1). These participants were recruited from the DELCODE study. DELCODE is an observational longitudinal memory clinic-based multicenter study in Germany. The aim of this still ongoing study is to enroll subjects with SCD, MCI patients, AD dementia patients, control subjects without subjective or objective cognitive decline and first degree relatives of patients with a documented diagnosis of AD dementia.

**Table 1:** Clinical and demographic characteristics of HC individuals and SCD patients.

	HC	SMC	p-value
N	134	111	
Age (y)	68.5 (5.0)	71.3 (5.6)	<0.001
Gender (m/f)	80/54	60/51	0.374
Education (y)	14. 9 (2.7)	14.7 (3.1)	0.450
MMSE	29.5 (0.9)	29.2 (1.0)	0.285
CDR	0.0 (0.0)	0.2 (0.2)	<0.001
LogMem I	15.1 (3.6)	14.6 (3.8)	0.960
LogMem II	13.9 (3.6)	13.1 (3.9)	0.585
Plasma tau	3.6 (1.7)	3.4 (1.2)	0.137

Note: Values are expressed as mean (standard deviation).

N: number; HC: healthy control individuals; SCD: patients with subjective cognitive decline; m/f: male/female; MMSE: Mini Mental State Examination; CDR: Clinical Dementia Rating Scale global score; LogMem: Logical memory subtest (I or II) of the Wechsler Memory Scale.

All participants underwent MMSE-scoring^12^, clinical assessment of cognitive status by means of the CDR scale^13, 14^, and the logical memory subtest of the Wechsler Memory Scale (i.e. immediate [LogMem I] and delayed story recall [LogMem II].

SCD was defined if participants were cognitively unimpaired and stated to have decline in cognitive functioning unrelated to an event or condition explaining the cognitive deficits according to recent research criteria^11^.

HC individuals never reported SCD and had no history of neurological or psychiatric disease or any sign of cognitive decline. Aβ42, tau and p-tau181 CSF levels were measured in the central lab of the DZNE in Bonn. Cut-offs for normal and abnormal concentrations of Aß42 (<600 pg/ml) and of the ratio Aß42/Aß40 (<0.09) were derived from the literature, which applied the respective assays^15^. For tau (>470 pg/ml) and p-tau181 (57 pg/ml) we used cut-offs established locally (Bonn) based on clinical and non-impaired control samples. In addition, we defined an abnormal Aβ42/tau ratio according to the formula of Hulstaert^16^ (Aβ42/[240 + 1.18 × tau] < 1), which has been shown to be a useful indicator of AD pathology^17^.

Experimental protocols described in the present study have been approved by the Ethik-Kommission an der Medizinischen Fakultät der Eberhard-Karls-Universität und am Universitätsklinikum Tübingen. All other aspects of the study have been approved by the institutional review boards for each of the participating sites in the DELCODE study. All methods were performed in accordance with the relevant guidelines and regulations. All participants provided written, informed consent.

### Blood sampling

Blood was obtained in the morning (9.00–10.00 A.M.; in the fasting state). Venous blood was collected in EDTA plasma tubes. EDTA plasma samples were centrifuged for 15 minutes at 10.000 g within 30 minutes of collection. Samples were aliquoted and stored at −80 °C before analysis.

### Simoa analysis

Plasma tau levels were determined using ultra-sensitive, single-molecule array (Simoa) technology^18^. Measurements were performed at the Natural and Medical Sciences Institute using the Human Total Tau 2.0 kit (Quanterix, Lexington, USA) following the instructions of the kit manual. All samples were measured in a final 1:4 dilution.

### CSF AD biomarker assessment

AD biomarkers were determined using commercially available kits according to vendor specifications (V-PLEX Aβ Peptide Panel 1 (6E10) Kit, K15200E and V-PLEX Human Total Tau Kit, K151LAE (Mesoscale Diagnostics LLC, Rockville, USA), and Innotest Phospho Tau(181P), 81581, Fujirebio Germany GmbH, Hannover, Germany).

### Data analysis

All statistical analyses were carried out using the statistical analysis software package SPSS (version 24). The data are presented as mean ± standard deviation (SD). Significance for the results was set at P < 0.05. Continuous variables were tested for normal distribution with the Kolmogorov-Smirnov test. Levene’s test served to assess homogeneity of variances. We used the Pearson chi-square test to detect group differences in gender distribution and the nonparametric Mann-Whitney U-test to detect group differences in CDR scores. Group differences in age and education were assessed using one-way ANOVA.

Differences between HCs and SCD in global cognition (MMSE), LogMem I and II, as well as Aβ42, Aβ42/40 ratio, CSF tau/Abeta42 ratio, tau and p-tau181 (if available), and plasma tau levels were assessed using one-way analyses of covariance (ANCOVA) controlling for age, gender, and education. Differences in tau plasma levels between normal HC and abnormal SCD according to different Aβ42 cut-off values (i.e. <600 pg/ml; on the basis of the Formula of Hulstaert^16^) and an Aβ42/40 ratio (i.e. <0.09) were evaluated using using one-way ANCOVA controlling for age, gender, and education.

Linear regression analysis were run to determine the relationship between tau plasma levels and age as well as with biochemical biomarkers (CSF levels of Aβ42, tau and p-tau181) or psychometric parameters (MMSE, LogMem I and II) controlling for age, gender, and education.

## Results

### Patients with SCD and HCs

Patients with SCD revealed significantly higher age compared to HCs (F[1,244] = 17.450; p < 0.001). CDR global score was significantly lower in HCs compared to SCD patients (p < 0.001). Gender (p = 0.374) and years of education (F[1,244] = 0.571; p < 0.450) were comparable between both groups. Additionally, MMSE (F[1,240] = 1.146; p < 0.285) scores as well as LogMem I (F[1,240] = 0.002; p < 0.960) and LogMem II (F[1,240] = 0.299; p < 0.585) subtest performance of the Wechsler Memory Scale did not differ between HCs and patients with SCD after controlling for age, gender, and education.

**Figure 1:**
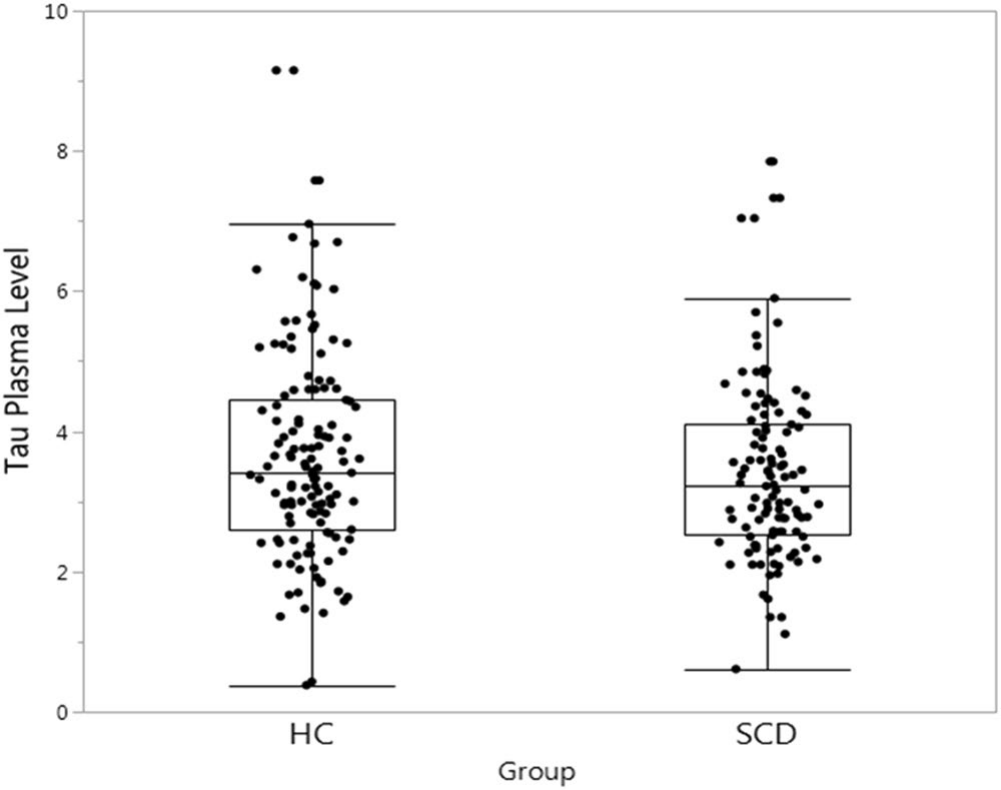
Plasma tau in HC individuals and SCD patients.

We found no significant different tau plasma levels (F[1,240] = 2.227; p = 0.137) in patients with SCD (3.4 pg/ml) compared with healthy controls (3.6 pg/ml) after controlling for age, gender, and education (Fig. 1). Additionally, tau plasma levels in SCD did not correlate with LogMem I (β = −0.084; 95% CI −0.774 to 0.262, p = 0.329) or LogMem II (β = −0.008; 95% CI −0.511 to 0.559, p = 0.929). However, there was a trend towards a negative association between tau plasma levels and MMSE (β = −0.176; 95% CI −0.276 to 0.001; p = 0.054).

All demographic, clinical, and neuropsychological parameters as well as tau plasma levels are displayed in Table 1.

### Study participants with available CSF

Participants with available CSF differed significantly in age (F[1,93] = 10.501; p = 0.002) with lower mean age in HC individuals compared to patients with SCD. Gender was equally distributed between the investigated groups (p = 0.488). Years of education (p = 0.786) and MMSE (F[1,89] = 1.963; p = 0.165) did not differ significantly between the groups. Scores in the LogMem I (F[1,89] = 0.119; p = 0.731) or LogMem II (F[1,89] = 0.440; p = 0.167) subtest of the Wechsler Memory Scale did not differ between HCs and patients with SCD.

After controlling for age, gender, and education we found no significant different tau plasma levels (F[1,89] = 0.238; p = 0.627) in patients with SCD (3.4 pg/ml) compared with HCs (3.6 pg/ml). Additionally, CSF levels of Aβ42 (F[1,89] = 3.327; p = 0.072), CSF Aβ42/40 ratio (F[1,89] = 0.095; p = 0.785), CSF tau/Abeta42 ratio (F[1,89] = 0.939; p = 0.335), tau (F[1,89] = 0.560; p = 0.456) and p-tau181 (F[1,89] = 0.320; p = 0.573) did not differ between the groups.

All demographic, clinical, and neuropsychological parameters as well as tau plasma, and CSF levels of Aβ42, tau, and p-tau181 for HC and SCD with available CSF are displayed in Table 2. Tau plasma levels in all SCD participants with available CSF did not correlate with CSF levels of Aβ42 (β = 0; 95% CI −0.001 to 0.001, p = 0.663), tau (β = 0.001; 95% CI −0.004 to 0.001, p = 0.298), and p-tau181 (β = 0.011; 95% CI −0.027 to 0.005, p = 0.171) after controlling for age, gender, and education.

**Table 2:** Clinical and demographic characteristics of HC individuals and SCD patients with available CSF levels of Aβ42, Ab42/40 ratio, tau, p-tau181, and CSF tau/Aβ42 ratio.

	HC	SMC	p-value
N	50	45	
Age (y)	68.0 (5.0)	71.3 (4.8)	0.002
Gender (m/f)	28/22	22/23	0.488
Education (y)	14.7 (2.8)	14.9 (3.4)	0.786
MMSE	29.5 (0.8)	29.2 (0.9)	0.165
CDR	0.0 (0.1)	0.2 (0.3)	<0.001
LogMem I	15.5 (3.6)	14.9 (3.4)	0.731
LogMem II	14.9 (3.9)	13.8 (3.3)	0.509
Plasma tau	3.6 (1.3)	3.4 (1.2)	0.627
CSF Aβ42	890.0 (322.9)	747.7 (329.3)	0.072
CSF Aβ42/40 ratio	0.098 (0.02)	0.093 (0.03)	0.758
CSF tau	359.0 (158.9)	365.6 (156.6)	0.456
CSF tau/Aβ42 ratio	0.452 (0.3)	0.59 (0.4)	0.335
CSF p-tau181	51.2 (19.9)	50.9 (23.6)	0.573

Note: Values are expressed as mean (standard deviation).

N: number; HC: healthy control individuals; SCD: patients with subjective cognitive decline; m/f: male/female; MMSE: Mini Mental State Examination; CDR: Clinical Dementia Rating Scale global score; LogMem: Logical memory subtest (I or II) of the Wechsler Memory Scale; CSF: cerebrospinal fluid.

Subgroup analysis of HC individuals with normal Aβ42 levels (i.e. ≥600 pg/ml; n = 41) and SCD patients with abnormal Aβ42 levels (i.e. <600 pg/ml; n = 17) revealed no significant different tau plasma levels between both groups (HCs: 3.45 pg/ml vs. SCD: 3.29 pg/ml; p = 0.654). In abnormal SCD participants there were no significant correlations between tau plasma levels and CSF levels of Aβ42 (r = −0.082; p = 0.782), tau (r = −0.413; p = 0.142), and p-tau181 (r = −0.226; p = 0.436) after controlling for age, gender, and education.

According to the Formula of Hulstaert^16^ no significant differences in tau plasma levels could be detected between normal HC individuals (3.47 pg/ml; n = 43) and SCD patients (3.31 pg/ml; n = 16; p = 0.642; Table 3). Tau plasma levels in abnormal SCD participants did not correlate with CSF levels of Aβ42 (β = 0.001; 95% CI −0.007 to 0.004, p = 0.527), tau (β = 0.003; 95% CI −0.006 to −0.001, p = 0.393), and p-tau181 (β = 0.019; 95% CI −0.038 to −0.001, p = 0.343) after controlling for age, gender, and education.

**Table 3:** Plasma tau levels of CSF negative (i.e. above cut-off) healthy controls (normal HC) and CSF positive (i.e. below cut-off) SCD patients (abnormal SCD) according to different cut-off levels using Aβ42, tau, and p-tau181.

Cut-off definition	normal HC above cut-off	abnormal SCD below cut-off	p-value
Hulstaert-formula (Aβ42/tau ratio; cut-off <1) no.	43	16	
Plasma tau	3.47 (1.1)	3.31 (1.3)	0.646
CSF Aβ42 (cut-off <600 pg/ml) no.	43	17	
Plasma tau	3.45 (1.3)	3.29 (1.1)	0.654
CSF Aβ42/40 (cut-off <0.09) no.	36	17	
Plasma tau	3.49 (1.2)	3.19 (0.9)	0.366

Note: Values are expressed as mean (standard deviation) or number (no.) above /below cut-off.

By using the Aβ42/40 quotient no significant differences in tau plasma levels between normal HC (i.e. Aβ42/40 ≥ 0.09 ratio; 3.49 pg/ml; n = 36) and abnormal SCD (i.e. Aβ42/40 < 0.09 ratio; 3.19 pg/ml; n = 17) could be detected (p = 0.366; Table 3). There were no significant associations between tau plasma levels and CSF levels of Aβ42 (β = 0.002; 95% CI −0.005 to 0.002, p = 0.316), tau (β = 0.003; 95% CI −0.005 to 0.000, p = 0.268), and p-tau181 (β = 0.014; 95% CI −0.032 to 0.005, p = 0.129) after controlling for age, gender, and education in abnormal SCD (results not shown).

## Discussion

As main finding of the present study, SCD patients showed no significantly different tau plasma levels in comparison with cognitively healthy controls in the whole study cohort and in the subgroup with available CSF. Given that SCD patients did also not show significantly different tau and p-tau181 CSF levels compared with healthy controls in the subgroup with available CSF, this finding was not surprising. However, even if different cut-off values of CSF Aβ42 and ratios with tau and Aβ40 were used to classify HCs with normal CSF values (i.e. Aβ42 above cut-off) and SCD patients with abnormal CSF values (i.e. Aβ42 below cut-off), tau plasma levels did not differ between these groups. Our results validate and extend the findings of the recently published BioFINDER (Biomarkers for Identifying Neurodegenerative Disorders Early and Reliably) study at Lund University, Sweden, examining tau plasma levels in 174 SCD patients and 274 healthy controls^5^. This study also failed to demonstrate increased tau plasma levels in SCD patients. Although negative, this consistent result of two independent European studies is important as it indicates that tau plasma levels are not a useful diagnostic measure for the pre-MCI stage of SCD. It is noteworthy that plasma tau peptides did not correlate with CSF tau peptides in SCD patients. This lack of correlation between tau levels in plasma and CSF is in line with the findings of previous studies^5, 6^. This suggests that tau levels are affected by different factors in both biofluids. Tau may derive from different sources in both body fluids, or the analysis in plasma might be influenced by unknown factors at lower levels, since a correlation has been reported for higher levels^5^.

Although there was no increased tau or p-tau in the SCD group compared to HC individuals, a statistical trend towards lower Aß42 concentration in SCD patients compared to HCs was observable. This might suggest that earliest cognitive changes (i.e. SCD) may already occur in the stage of Aß42 accumulation in the absence of significant neurodegeneration^19^.

Previous studies showed increased tau plasma levels in patients with AD^4–6^, in a group of patients with AD and mild cognitive impairment (MCI) due to AD^7^ and in MCI due to AD^4^, but not in clinically classified MCI groups converting or not to AD during follow-up^5, 6^. Taken together, our own result and these findings in literature indicate that plasma tau is a late marker of neurodegeneration, requiring substantial injury before increasing to abnormal levels at the transition from MCI to dementia stage of AD.

As limitation of the study, CSF was not available in all study participants but only in a subgroup of 45 SCD patients and 49 healthy controls. In addition, currently no follow-up data were available. Additionally, the Formular of Hulstaert and cut-off levels of Abeta and tau are not widely accepted standards for peripheral biomarker analysis. Positive outcomes would reinforce the values of the formula and cut-off levels, but negative outcomes are not informative.

In conclusion, plasma tau is not altered in the examined cohort of subjects at increased risk for AD. In addition, the lack of correlation between tau in plasma and CSF in the examined cohort suggests that tau levels are affected by different factors in both biofluids.
